# Functional characterization of a novel c.614-622del rhodopsin mutation in a French pedigree with retinitis pigmentosa

**Published:** 2012-03-02

**Authors:** Cécilia Maubaret, Maria Kosmaoglou, Sancy Low, Christina F. Chakarova, Samuel Bidot, Christel Thauvin-Robinet, Anthony G. Robson, Naushin Waseem, Michael E. Cheetham, Shomi S. Bhattacharya

**Affiliations:** 1 UCL Institute of Ophthalmology, London, United Kingdom; 2 Moorfields Eye Hospital, London, United Kingdom; 3 Hôpital Des Enfants, Dijon, France

## Abstract

**Purpose:**

To identify and functionally characterize the mutation responsible for autosomal dominant retinitis pigmentosa (adRP) in a large, six-generation French family.

**Methods:**

Twenty individuals from this family participated in the genetic investigation. Six affected and 14 unaffected individuals from three-generations were available for linkage analysis using microsatellite markers flanking the rhodopsin (*RHO)* gene. A two-point logarithm of odds (LOD) score calculation was undertaken using GENEMARKER and MLINK software. Sanger sequencing of *RHO* was performed. Cellular localization of the mutant protein was performed by transforming SK-N-SH cells with pEGFP-N1-Rho, pEGFP-N1-Rho(P23H), and pEGFP-N1-Rho(c.614–622del).

**Results:**

The proband had nyctalopia, visual field constriction, peripheral bone spicule pigmentation of the fundus, central acuity (6/24 RE; 6/12 LE) at 55 years of age. Linkage analysis of this family suggested *RHO* as a possible candidate since the flanking marker D3S1292 yielded a LOD score of 2.43 at θ=0. Cloning of an exon 3 PCR product and direct sequencing of single clones identified a novel deletion in the third exon of *RHO,* c.614–622del (p.Y206-F208del). The deleted mutant protein localized to the endoplasmic reticulum and formed inclusion bodies.

**Conclusions:**

This novel deletion in exon 3 of the *RHO* gene, c.614–622del results in a classical form of adRP in a multi-generation French family. Protein expression analyses confirmed that the deletion led to protein misfolding and suggest this is a class II mutation, similar to P23H, the most common class II mutation seen in North America.

## Introduction

Retinitis pigmentosa (RP) has a prevalence of about 1:4,000 and is the most frequent form of inherited peripheral retinal degeneration [1]. Multiple modes of inheritance have been reported, and over 45 causative genes have now been identified (RetNet). Rhodopsin *(RHO)* mutations are one of the most common causes of RP in humans (8%–10%) [2,3]. They account for a quarter of all autosomal dominant RP (adRP) cases (RetNet), but autosomal recessive inheritance has also been described [4,5]. Mutations in *RHO* are associated with a range of phenotypes including mild to severe RP, sector RP, and rare cases of congenital stationary night blindness [5–7].

In the early 1990s, Sung et al. [8] and Kaushal et al. [9] proposed a classification of *RHO* mutations according to the localization of the mutant protein and its capacity to bind 11-*cis*-retinal. Class I mutations predominantly occur in the C-terminal of the protein, are able to bind to 11-*cis*-retinal, and form a functional chromophore. In contrast, class II mutations, which occur in the intradiscal, transmembrane or cytoplasmic domains of the protein, result in misfolding of the protein and make it unable to bind to 11-cis-retinal. Class II mutations are more common. In a recent review, Mendes et al. [10] proposed an extended classification of *RHO* mutations, expanding *RHO* mutations into five further subgroups according to their intracellular and biochemical behavior. According to this nomenclature, the most common cause of adRP in North America, P23H, is a class II mutation. The mutant protein is retained in the endoplasmic reticulum (ER) and does not reconstitute with 11-*cis-*retinal.

In this study, we report a novel in-frame *RHO* deletion identified in a large, French family with adRP. The cellular localization of the deleted mutant protein is presented and compared with the common P23H mutation.

## Methods

### Recruitment of patients and phenotype analysis

**Figure 1:**
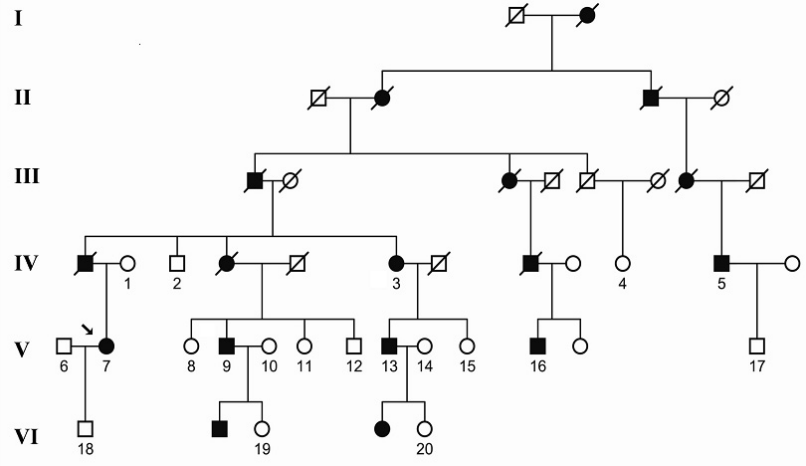
Pedigree of the French family recruited in Dijon. Subjects affected by autosomal dominant retinitis pigmentosa are indicated by solid (black) symbols, while unaffected individuals are indicated by open (white) symbols. Slashed symbols represent deceased family members. Subjects for whom samples were available for molecular analysis are labeled with an Arabic numeral. The proband is indicated by an arrow.

A six-generation French family with 17 members affected with adRP participated in this study. Informed consent for genetic investigation was obtained from 20 subjects (6 affected and 14 unaffected; Figure 1) in accordance with guidelines established by the Declaration of Helsinki. This project was approved by the Institutional Review Board at Hôpital des Enfants, Dijon, France. A full medical history was captured at the time of the first hospital visit and ophthalmic examinations were performed for all available family members. The phenotype of the family is represented by the proband (individual V.7) in this report. Visual acuity testing, color fundus photography, and automated suprathreshold testing using the full field 120 program of the Humphrey Field Analyzer (Zeiss, Germany) was performed. Genomic DNA was obtained using Nucleon DNA Isolation Kits for Mammalian Blood according to the manufacturer’s instructions (Tepnel Life Sciences, Manchester, UK).

### Mutation detection

#### Linkage analysis

Microsatellite markers flanking known genes for adRP were selected from the ABI Prism Linkage Mapping Set v 2.5 (Applied Biosystems, Foster City, CA) and the Ensembl database. Sequences are available on request. PCRs were performed with the Absolute QPCR kit, accordingly to the manufacturers’ instructions (ABgene, Epsom, UK). The resultant PCR products were diluted in HiDi formamide containing GeneScan^TM^-500 LIZ ® fluorescent dye (Applied Biosystems, UK). After denaturation, the samples were loaded on a DNA sequencer (model 3730; Applied Biosystems [ABI], Cheshire, UK) and the genotyping calls and Mendelian error checks were performed with the GENEMARKER software (Biogene, Cambridge, UK). Two point LOD scores were calculated using the MLINK program [11] with a parametric linkage model of autosomal dominance, penetrance of 0.9, and with a disease gene frequency of 0.0001.

#### Direct DNA sequencing

The five exons and flanking introns of the *RHO* gene were amplified using primers previously described [12] and Gold Taq polymerase (ABI). PCR products were sequenced using a BigDye terminator sequencing kit ver.1.1 (ABI) and the ABI 3730 DNA Sequencer.

#### Cloning of the mutated allele

An allele-specific cloning and sequencing approach was used to precisely characterize the deletion. Briefly, exon 3 of *RHO* was amplified from genomic DNA by PCR and the purified amplicon was ligated, by means of a TA-ligation method, into the TA-cloning vector pGEM-T (Promega, Southampton, UK), and was subcloned into JM109 competent cells (Stratagene, La Jolla, CA) under normal culture conditions. Plasmid Minipreps were prepared (Sigma-Aldrich, Dorset, UK) and sequenced using the T7 and SP6 primers.

### Localization of the mutant proteins in SK-N-SH cells

The plasmids, pEGFP-N1-Rho and pEGFP-N1-Rho(P23H), encoding rod opsin-GFP fusion proteins have been described previously [13]. Plasmid pEGFP-N1-Rho was used as the template for generation of the c.614–622del (p.Y206-F208del) mutant using site specific primers (5′-ACA ATG AGT CGT TCG TCA TCG TGG TCC ACT TCA TCA TCC C-3′ and 5′-GGG ATG ATG AAG TGG ACC ACG ATG ACG AAC GAC TCA TTG T-3′) and using the QuikChange site-directed mutagenesis kit according to the manufacturer's instructions (Stratagene, La Jolla, CA). The pEGFP-N1-Rho(Y206-F208del) plasmid was sequenced to detect any PCR artifacts and to confirm the presence of the mutation. SK-N-SH human neuroblastoma cells were acquired from the European Collection of Cell Cultures and were transfected at low passage numbers (<P20) using Lipofectamine PLUS and according to the manufacturer’s instructions (Life Technologies, Paisley, UK). The subcellular distribution of RHO (Y206-F208del) in SK-N-SH neuroblastoma cells was compared to wild-type and P23H mutant by visualizing GFP with epifluorescence microscopy (Nikon Eclipse 80i, Derby, UK). For morphological analyses, three groups with approximately 100 transfected cells in each were counted and the distribution of WT opsin-GFP, P23H opsin-GFP, and Y206-F208del opsin-GFP in transfected cells was classified either as predominantly plasma membrane, predominantly ER, or as containing inclusions. Counts were analyzed using the ANOVA test (ANOVAR) to compare the means of two samples. Images were taken with a Zeiss LSM 510 confocal microscope (Welwyn Garden City, UK).

## Results

### Clinical phenotype

**Figure 2:**
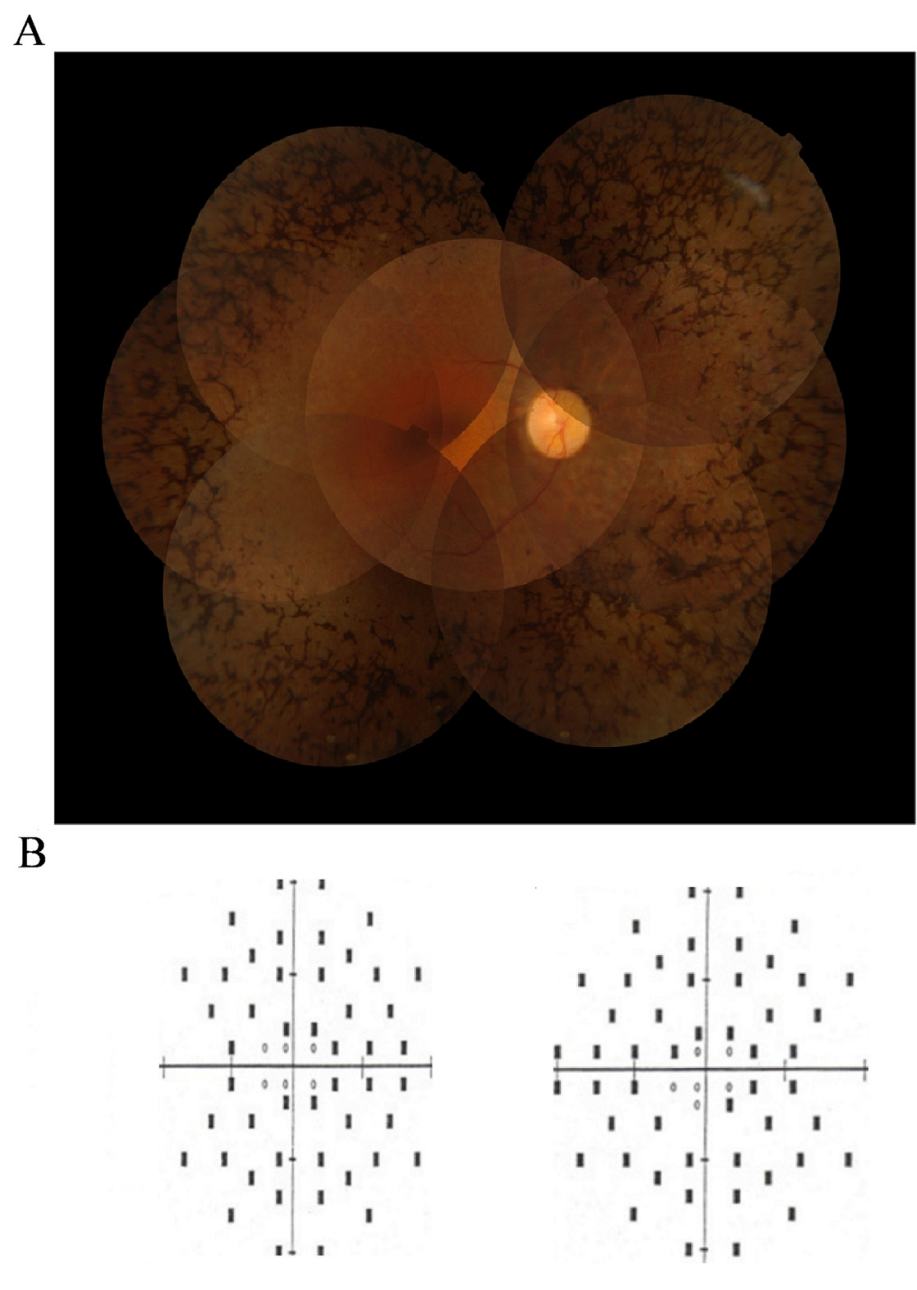
Clinical information on the proband. **A**: Fundus photography of the right eye shows classical bony-spicules, attenuated vessels, and waxy pale optic discs. **B**: Results of a120-point visual field screening showing severe constriction of both visual fields. In this automatic visual field examination (screening test), visual stimuli were presented in different points of the visual field (throughout a virtual map shown here). White circles (in the center) represent stimuli detected by the proband, whereas black rectangles correspond to undetected stimuli.

The RP in the family segregates in an autosomal dominant pattern (Figure 1). The phenotype observed was typical with bone-spicule pigmentation, decreased visual acuity, attenuation of the retinal blood vessels, and pale optic discs and night blindness (Figure 2A). The proband (individual V.7) was 55 years old at her last clinical examination. Her visual acuity was 6/24 (right eye) and 6/12 (left eye). She had already undergone bilateral cataract surgery. Severe constriction of the visual field was recorded on the 120-point Humphrey screening test (Figure 2B). Her husband and son had normal fundal examinations. The first cousin of the proband, individual V.9, had bone spicule pigmentation of his fundus at the last follow-up visit at 50 years of age, with macular atrophy resulting in visual acuities of 6/60 in the right eye and 6/18 in the left eye. He was registered partially sighted at that visit. His affected son developed early peripheral retinal signs suggestive of RP at the age of 11 years, and was diagnosed at the age of 15 years. He maintained good visual acuities of 6/9 in the right eye and 6/12 in the left eye at the age of 21 years. Individual V.11 (visual acuities of 6/6 bilaterally), and VI.20 had a normal ophthalmic examination. Individual V.16 had moved away from France but was known to have lost sight, presumably from RP. The attending family members corroborated the family history and affected status of those who send blood samples but did not attend for clinical examination.

### Linkage to chromosome 3

The search for the causal mutation segregating the family began by testing known adRP genes and loci using a targeted and cost-effective method of linkage analysis and commercially available microsatellite markers. The linkage analysis of the 20 participants (numerically indicated in Figure 1) generated a two-point LOD score of 2.43 at θ=0 for marker D3S1292, located 2Mb 3′of the *RHO* gene. Although not statistically significant, this result was suggestive of linkage to the *RHO* locus as it is the most common causative gene in adRP families. All five exons of the *RHO* gene were sequenced for individuals 9 and 13. A sequence aberration was detected in exon three for both individuals, but was difficult to identify properly, indicating it could be a deletion or an insertion. To characterize this change, we isolated the sequences by allele-specific cloning.

### Nine base-pair deletion in exon 3 of the *RHO* gene

**Figure 3:**
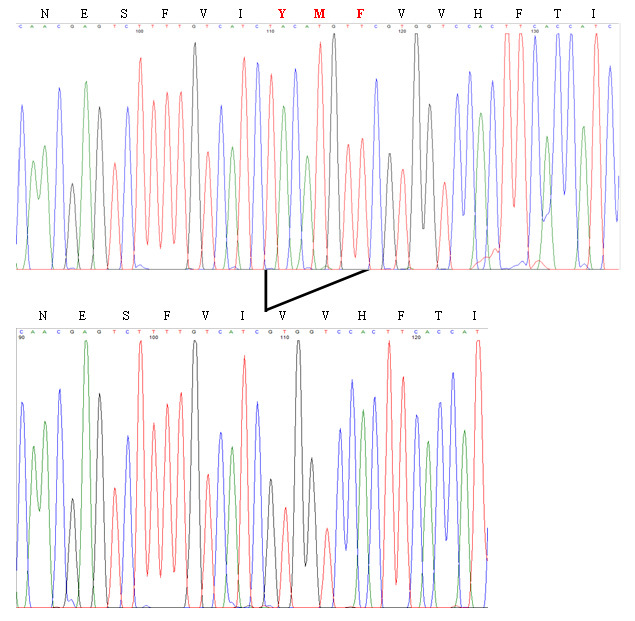
*Rhodopsin* mutation in exon 3. Detection of the novel c.614–622del (p.Y206-F208del) mutation in the *RHO* gene from a sequence analysis after cloning of both alleles. The electropherogram representing the normal sequence is shown on top while the deleted one is presented below.

Cloning of both PCR fragments (wild type and mutant) from individual 9 identified a novel mutation in the *RHO* gene: a nine base-pair deletion from nucleotide 614 to nucleotide 622 in exon three, c.614–622del (Figure 3). The in silico analysis of the mutant sequence predicts deletion of amino acid 206 to 208, which are tyrosine, methionine, and phenylalanine (p.Y206-F208del). The mutation co-segregated with the disease: it was identified in all six participants with a positive history or clinical signs of RP, but none of the unaffected individuals were carriers of this change.

### Localization of the mutant protein in SK-N-SH cells

**Figure 4:**
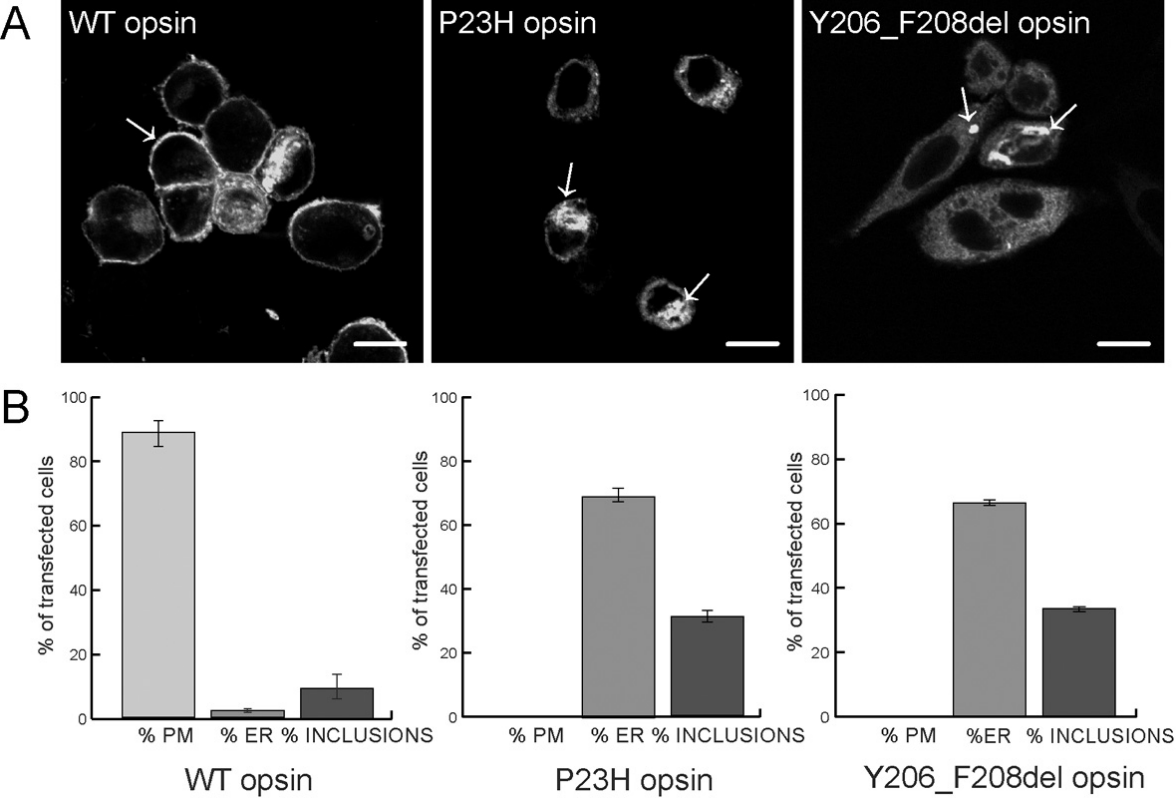
Trafficking studies of the mutant protein. **A**: Subcellular localization of the WT opsin-GFP, P23H opsin-GFP and the novel c.Y206-F208del opsin-GFP mutation in SK-N-SH neuroblastoma cells. The arrows indicate plasma membrane localization for the WT opsin and inclusion bodies for both mutant proteins. The scale bar represents 10 μm. **B**: Quantification of the predominant subcellular localization in SK-N-SH neuroblastoma of the WT opsin-GFP, P23H opsin-GFP, and Y206-F208del opsin-GFP fusion proteins 24 h after transfection. Three batches of 100 cells were counted and predominant localization of the opsin in the plasma membrane (PM), endoplasmic reticulum (ER), or the presence of an inclusion were graded. Error bars represent ±2 standard errors.

The neuroblastoma derived cell line, SK-N-SH, have been used previously to study the heterologous expression of mutant rod opsins [14–16], therefore, SK-N-SH cells were transfected with pEGFP-N1-Rho, pEGFP-N1-Rho(P23H), or pEGFP-N1-Rho(c.614–622del), encoding wild-type (WT) opsin-GFP, P23H opsin GFP, and Y206-F208del opsin-GFP, respectively. Rod opsin expression has been investigated in a range of cultured cell types and it is well documented that WT rod opsin translocates to the plasma membrane in the absence of an outer segment [8,9,13–16]. In the present experiment, WT opsin-GFP localized predominantly to the plasma membrane with some staining in the ER and Golgi network, indicating the normal biogenesis and transportation of the protein to the membrane (Figure 4A). By contrast, the P23H opsin-GFP and Y206-F208del opsin-GFP mutants were not trafficked to the plasma membrane and accumulated intracellularly. The pattern of Y206-F208del rod opsin-GFP fluorescence was reticular and was excluded from the nucleus in a manner similar to the pattern for P23H rod opsin, which is ER retained [13–16], suggesting ER retention of the in-frame deletion mutant. Both mutants also formed intracellular inclusion bodies (indicated by arrows). The effect of the in-frame deletion mutation on the proportion of opsin-GFP expressing cells with inclusions was quantified (Figure 4B). Approximately 9% of cells expressing wild-type opsin-GFP contained inclusions 24 h after transfection and 2% were predominantly retained in the ER. The incidence of inclusions or ER retention rose to 31% and 69%, respectively, for cells expressing P23H mutant rhodopsin-GFP, in accordance with previously published data [14–16]. The Y206-F208del opsin-GFP mutant resembled the P23H mutant with approximately 34% of cells expressing Y206-F208del rod opsin-GFP bearing inclusions 24 h after transfection and 66% with an ER pattern of fluorescence. Y206-F208del rod opsin-GFP was not detected on the plasma membrane in any transfected cells.

## Discussion

In this publication, we report a novel nine base-pair (bp) deletion in a six-generation French family presenting with classical adRP. Prior to this study, 10 small deletions (Retinal International database) had been reported in the *RHO* gene, out of which, four are multiples of three nucleotides and therefore do not create frameshift. The c.614–622del (p.Y206-F208del) mutation reported here is the first small deletion found in the third exon of the gene. Keen et al. [17] described an in-frame 12 bp deletion in exon 1, c.204–215del (p.R68-N72del) in a small English pedigree, who presented with a mild phenotype and relatively good visual field preservation in the fourth decade of life. Inglehearn et al. [12] reported a family with a three bp deletion in exon 4, c.768–770del (p.I255del). Affected individuals from this family reported symptoms of RP early in life but had good visual field preservation in the fourth decade of life and good visual acuity was retained until the seventh decade of life. The phenotypes described for those families support our present findings, suggesting that in-frame small deletions induce a classical, moderate form of RP which progresses slowly with age. Individuals V.7 and V.9 of the family carrying the novel c.614–622del (p.Y206-F208del) that we presented here maintained moderate visual function into the fifth decade of life. It is worth noting that Farrar et al. [18] reported a missense mutation resulting in a methionine to arginine change at codon 207, which is the middle codon of the deletion in our family (codons 206–208). The phenotype reported for that family had a very early onset (within the first decade of life), and ERG cone responses were only retained until the third decade. This small group of families displays an older age of visual preservation and less severe clinical characteristics in small in-frame deletions (including multiples of three) compared to the family with the p.M207R missense mutation. There is significant clinical and genetic heterogeneity in families with *RHO*-associated RP.

The novel deletion described in this report, c.614–622del causes the deletion of Y206, M207, and F208. Throughout the 53 species reported in the ensemble database with complete rhodopsin sequences (Ensembl database), the sequence YMF is conserved in 50 species, suggesting that the three amino acids considered separately are necessary for the function of the protein. In the wild type sequence, Y206, M207, and F208 are part of the fifth transmembrane helices of the *RHO* protein. Tyrosine and phenylalanine are amino acids containing a large aromatic chain. Usually, aromatic chains allow secondary folding of the protein, so it is likely that the absence of Y206 and/or F208 disrupts the transmembrane helix, leading to partial or complete misfolding of the rhodopsin protein.

According to the classification of the *RHO* mutations made by Sung et al. [8], Kaushal et al. [9] and more recently by Mendes et al. [10], class II mutations result in misfolding of the protein. Class II mutations are the most common since the rhodopsin protein is particularly sensitive to misfolding [19], which compromises the normal biosynthetic activity of the secretory pathway in the inner segment. The most common and, therefore, most described class II mutation in North America is p.P23H. In animal models of the p.P23H mutation, the shortening of outer segments has also been observed [20].

c.614–622del (p.Y206-F208del) results in the deletion of part of the fifth transmembrane domain of the rod opsin protein and because of the expected alteration in secondary folding induced by deletion of Y206 and F208, the c.614–622del (p.Y206-F208del) deletion presented here was expected to induce misfolding of the protein and retention in the ER, and therefore to be a class II mutation. To test this hypothesis, we transfected SK-N-SH neuroblastoma cells and compared the cellular localization of p.Y206-F208del to WT and p.P23H rod opsin-GFP constructs. Morphological observation revealed that both mutant proteins accumulate in the ER and form inclusion bodies. The p.Y206-F208del deletion reported here also formed intracellular inclusions suggesting it is aggregation prone. Hence, similarly to p.P23H [10,13], the novel p.Y206-F208del mutant protein belongs to class II *RHO* mutations [10]. Seven other mutations in the fifth transmembrane helix of rhodospin have been identified. Two of the mutations (p.H211F and p.C222R; Ensemble database) have been characterized as class II mutations, confirming that mutations in transmembrane domains are likely to induce misfolding of the protein.

The intracellular fate of mutant rhodopsin molecules is a very important factor for gaining a full appreciation of the consequences and mechanism(s) of *RHO*-associated RP. Identification and characterization of *RHO* mutations provide the potential to enhance our phenotype-genotype understanding, and improve molecular diagnostic tools such as microarray SNP-chips [21]. The combined use of clinical, genetic, and functional data including in vitro expression experiments shows promise as an approach to improve our understanding of *RHO*-associated pathophysiology.
